# A lipidomic dataset for epidemiological studies of acute myocardial infarction

**DOI:** 10.1016/j.dib.2024.110925

**Published:** 2024-09-12

**Authors:** Cecilia Castro, Eric L. Harshfield, Adam S. Butterworth, Angela M. Wood, Albert Koulman, Julian L. Griffin

**Affiliations:** a Rowett Institute, University of Aberdeen, Foresterhill, Aberdeen, AB25 2ZD, UK; b Department of Clinical Neurosciences, University of Cambridge, Addenbrooke's Hospital, Cambridge, CB2 0QQ, UK; c British Heart Foundation Cardiovascular Epidemiology Unit, Department of Public Health and Primary Care, University of Cambridge, Cambridge, UK; d Victor Phillip Dahdaleh Heart and Lung Research Institute, University of Cambridge, Cambridge, UK; e British Heart Foundation Centre of Research Excellence, University of Cambridge, Cambridge, UK; f National Institute for Health and Care Research Blood and Transplant Research Unit in Donor Health and Behaviour, University of Cambridge, Cambridge, UK; g Health Data Research UK Cambridge, Wellcome Genome Campus and University of Cambridge, Cambridge, UK; h Cambridge Centre of Artificial Intelligence in Medicine, UK; i British Heart Foundation Data Science Centre, Health Data Research UK, London, UK; j Institute of Metabolic Science-Metabolic Research Laboratories, University of Cambridge, Addenbrooke’s Hospital, Cambridge CB2 0QQ, UK

**Keywords:** Lipidomics, Mass spectrometry, Direct infusion, Epidemiology, Coronary heart disease

## Abstract

Understanding the cause of coronary heart diseases relies on the analysis of data from a range of techniques on an epidemiological scale. Lipidomics, the identification and quantification of lipid species in a system, is an omic approach increasingly used in epidemiology. The altered concentration of lipids in plasma is one of the recognised risk factors for these diseases. An important first step in the analysis is to profile lipids in healthy volunteers at an epidemiological level to understand how the geneome influences risk factors; for this reason we made use of the control samples within a bigger case-control sample collection in Pakistan from patients with first acute myocardial infarctions. After extraction, the samples were infused into a Thermo Exactive Orbitrap, without any up-front chromatographic separation. The use of direct infusion allowed fast experiment, facilitating the analysis of large sets of samples. The raw data were processed and analysed using scripts within R, to extract all the meaningful information. The data set originated from this study is a valuable resource to both increase our knowledge in lipid metabolism associated with myocardial infarction, and test new methods and strategy in analysing big lipidomic data sets.

Specification Table

**Table:** 

Subject	Omics: Lipidomics.
Specific subject area	Lipidomics quantification from plasma of control patients within a case-control study of first-ever acute myocardial infarction.
Type of data	Table
Data collection	Non-fasting blood samples were drawn from each participant. The samples were extracted, using a protocol with 2.5:5:1 CH_3_OH:MTBE:H_2_O, then infused into an Exactive Orbitrap, using a Triversa Nanomate. The samples were carefully processed using in-house developed scripts in R to obtain the best data quality.
Data source location	The data were collected at the Medical Research Council Human Nutrition Unit, 120 Fulbourn Road, Cherry Hinton, Cambridge, CB1 9NL, UK.
Data accessibility	Repository name: Metabolights Data identification number: MTBLS4461 Direct URL to data: https://www.ebi.ac.uk/metabolights/editor/MTBLS4461/descriptors Instructions for accessing these data: Data are directly accessible.
Related research article	Eric L. Harshfield, Albert Koulman, Daniel Ziemek, Luke Marney, Eric B. Fauman, Dirk S. Paul, David Stacey, Asif Rasheed, Jung-Jin Lee, Nabi Shah, Sehrish Jabeen, Atif Imran, Shahid Abbas, Zoubia Hina, Nadeem Qamar, Nadeem Hayyat Mallick, Zia Yaqoob, Tahir Saghir, Syed Nadeem Hasan Rizvi, Anis Memon, Syed Zahed Rasheed, Fazal-ur-Rehman Memon, Irshad Hussain Qureshi, Muhammad Ishaq, Philippe Frossard, John Danesh, Danish Saleheen, Adam S. Butterworth, Angela M. Wood, and Julian L. Griffin An unbiased lipid phenotyping approach to study the genetic determinants of lipids and their association with Coronary Heart Disease risk factors. J Proteome Res. 18, 2397-2410 (2019).

## 1 Value of the Data

• This lipidomic data set provides a tool to start to define normal variation within a control population at risk of coronary heart disease on an epidemiological scale.
• Scientists studying heart disease can take advantage of this data set, together with more broadly scientists interested in lipid levels in blood for other conditions. Furthermore, bioinformaticians developing new tools for data processing and analysis can use them.
• It can be used as a comparison data set with other control populations from different geographical regions or patient profiles, as well as a comparison with patients from specific disease cohorts.

## 2 Background

Coronary heart disease (CHD) is the world leading cause for mortality and morbidity, irrespective of the average income of a country [1]. South Asians are particularly affected as CHD mortality and morbidity remain higher in immigrant South Asians living in western regions compared with native western populations [2] and evidence suggests that these populations are predisposed for CHDs due to a combination of hereditary and lifestyle factors [3,4]. To address the paucity of epidemiological resources available in that geographical area, and therefore help shed light on the CHD incidence, the Pakistan Risk of Myocardial Infarction Study (PROMIS) was established. This is a retrospective case-control study of first-ever acute myocardial infarction (MI) in patients from nine centres in urban Pakistan [5] and consists of around 16,700 cases and 18,600 controls. As dyslipidaemia, the altered concentration of lipids in blood plasma, is one of the recognised risk factors of CHD [6], the analysis of lipid profiles in PROMIS samples may shed light into how genetic risk factors modify the blood lipidome prior to overt disease.

In this report, we describe the lipidomic method applied to a subset of 5662 controls from PROMIS and the data generated from that. The specific protocol applied involves direct infusion high-resolution mass spectrometry (DIHRMS), which yields the intensities of several thousand of features within a specified mass-to-charge window in a non-targeted way. This allows the establishment of a baseline in lipid levels within a South Asian population, useful as a tool to establish changes in patients and to understand their origin. Its use, however, could go beyond this aspect, as it can also be used as a validation set for other lipidomic studies in South Asia or other parts of the world, or as a benchmark to test new tool to process and/or analyse direct infusion mass spectrometry data.

## 3 Data Description

**i_investigation.txt** A table containing summary basic information about the study submitted.
**s_MTBLS4461.txt** A table containing key information for each sample analysed within the study, to help in the analysis and interpretation of the results.
**a_MTBLS4461_DI-MS_positive_metabolite_profiling.txt** A table containing all the information about each sample run in the data set, including the processed intensity for each variable extracted.
**m_MTBLS4461_DI-MS_positive_metabolite_profiling_v2_maf.txt** A table containing key information about each variable extracted from the data set, including the assignment to lipid species.
**Raw files**. All the files obtained from the sample running, including blanks and QCs.

## 4 Experimental Design, Materials and Methods

**Sample collection.** Fulfilling all of the following criteria made patients aged 30–80 years admitted to the emergency rooms eligible for inclusion in the study: (1) sustained clinical symptoms suggestive of MI lasting longer than 20 min within the previous 24 h; (2) ECG changes of MI (i.e., new pathologic Q waves, at least 1 mm ST elevation in any 2 or more contiguous limb leads or a new left bundle branch block, or new persistent ST–T wave changes diagnostic of a non-Q wave MI); (3) confirmatory troponin-T measurements; and (4) no previous cardiovascular disease. As controls, individuals without a self-reported history of cardiovascular disease (whose ECG shows no changes consistent with a previous MI) concurrently in the same hospitals were identified. Exclusion criteria included (1) previous history of cardiovascular disease; (2) an infection that occurred in the previous 2 weeks; (3) documented chronic conditions, such as malignancy, any chronic infection, leprosy, malaria or other bacterial/parasitic infections, chronic inflammatory disorders, hepatitis or renal failure on past medical history; (4) pregnancy; or (5) refusal to give consent. Standardized procedures and equipment were used for anthropometry measurements, including height, weight, systolic blood pressure (SBP), and diastolic blood pressure (DBP). Non-fasting blood samples were obtained from each participant, centrifuged within 45 min of venipuncture, frozen, and transported on dry ice to Cambridge, UK. Major lipids and other standard biomarkers (e.g., total cholesterol, low-density lipoprotein cholesterol [LDL-C], high-density lipoprotein cholesterol [HDL-C], total triacylglycerols, hemoglobin A1c [HbA1c], apolipoprotein B, C-reactive protein) were measured for each sample. Samples were stored at −80 °C until use.

**Sample clinical information.** 5674 PROMIS participants, with genetic data and complete information on age, sex, ethnicity, recruitment centre, and date of survey completion, were selected for lipidomics analysis. Only controls (i.e., non-MI) were included to avoid possible confounding factors from the metabolic response of patients with a recent MI. The demographic and key characteristics of the analysed participants are included in the submitted data, in particular the sample table. The median age, sex representation and the average body mass index (BMI) mirrored the characteristics of the entire PROMIS study [7]. However, it is worth noting that PROMIS participants do not mirror the characteristics of the head of household in the general Pakistani population based on nationwide survey data obtained from the Demographic and Health Survey for Pakistan [8]. In fact PROMIS participants were older, a higher proportion consumed tobacco and were overweight [7].

**Sample extraction and preparation.** Adapting a method for open-profiling of lipids by DIHRMS [9,10], a protocol for the extraction of lipids was developed and then automated using an Anachem Flexus automated liquid handler (Anachem, Milton Keynes, UK). Groups of 96 samples, constituting one plate, were organised as follow: 15 µL each from eighty PROMIS samples, four blanks, and twelve quality control samples in 1.2 mL Cryovials. All the samples in each group were placed on the Flexus, then 100 µL of Milli-Q H_2_O was mixed. 100 µL of the mixture was transferred to a glass coated 2.4 mL deep well plate (Plate+, Esslab, Hadleigh, UK), and 250 µL of MeOH containing six internal standards (0.6 µM 1,2-di-O-octadecyl-snglycero-3-phosphocholine, 1.2 µM 1,2-di-O-phytanyl-sn-glycero-3-phosphoethanolamine, 0.6 µM C8-ceramide, 0.6 µM N-heptadecanoyl-D-erythro-sphingosylphosporylcholine, 6.2 µM undecanoic acid, 0.6 µM trilaurin) was added, followed by 500 µL of methyl tert-butyl ether (MTBE). After that, the plates were sealed (Corning aluminium microplate sealing tape [SigmaAldrich Company, UK]), shaken for 10 min at 600 rpm (4g), and then transferred to a centrifuge and spun for 10 min at 6000 rpm (4000g). After this step, two layers were present in each well, the aqueous one at the bottom and the organic layer on top. Using a 96-head microdispenser (Hydra Matrix, Thermo Fisher Ltd., Hemel Hempstead, UK) 25 µL of the organic layer were transferred to a glass coated 240 µL low well plate (Plate+, Esslab, Hadleigh, UK), then 90 µL of MS-mix (7.5 mM NH_4_Ac IPA:MeOH [2:1]) added finally the plate was sealed and stored at −20 °C until analysis.

**Direct infusion high-resolution mass spectrometry.** The samples were run on an Exactive Orbitrap (Thermo, Hemel Hempstead, UK), equipped with a Triversa Nanomate (Advion, Ithaca, US). The Nanomate infusion mandrel pierced the seal of each well then aspirated 5 µL of sample and an air gap (1.5 µL). The sample was dispensed using 0.2 psi nitrogen pressure, after pressing the tip against a fresh nozzle. A 1.2 kV voltage allowed sample ionization. The Exactive started data acquisition 20 s after the start of sample aspiration and acquired the data with a scan rate of 1 Hz,resulting in a mass resolution of 65 000 full width at half-maximum [fwhm] at 400 m/z. After 72 s of acquisition in positive mode the Nanomate and the Exactive switched over to negative mode, with a voltage of −1.5 kV. After further 66 s, the analysis was stopped, the tip discarded and the Nanomate proceeded to the of the following sample. The samples were kept at 15 °C throughout the analysis.

**Processing.** The quality of a sample was considered poor if there was no signal or the intensity of the total ion current was below 10^5^. For this reason, 259 samples necessitated a repeated analysis. Once the acquisition was successfully completed, the files were decompressed and converted to mzXML format using the “msconvert” tool in ProteoWizard [11]. For each infusion, an average spectrum was calculated from the user defined time window. The “xcms” R package 7 was used to average 50 spectra per mode using an m/z window of 185−1000 and a retention time window of 20−70 s for positive ionization mode and 95−145 s for negative ionization mode. Lipid identification was performed prior to data processing, analysing the pooled sample on a Velos Elite Orbitrap interfaced with an Advion Nanomate with the same ionization parameters used before. A combination of collision-induced disassociation (CID) and high-energy collision disassociation (HCD) fragmentation were used to fragment. Fragmentation patterns were matched using the LIPID Metabolites and Pathways Strategy (LIPID MAPS) database [12]. Peaks were identified and retained if they were within a ± 5 ppm window of the target m/z for known lipids present in LIPID MAPS. In particular, the following lipid subclasses were searched in positive ionization mode cholesterols (Chol), cholesteryl esters (CE), diacylglycerols (DG), triacylglycerols (TG), phosphatic acids (PA), lysophosphosphocholines (LysoPC), phosphatidylcholines (PC), phosphatidylethanolamines (PE), and sphingomyelins (SM); while in negative ionization mode, were ceramides (Cer), free fatty acids (FreeFA), PA, PC, PE, phosphatidylglycerols (PG), phosphatidylinositols (PI), phosphatidylserines (PS), and sphingomyelins (SM). The following adducts were considered: [M + H]^+^, [M + NH_4_]^+^, and [M + H − 2H_2_O]^+^ in positive ionization mode, and [M − H]^−^ and [M + acetate]^−^ in negative ionization mode. The CAMERA package [13] allowed the assessment of the isotope labelling. A more accurate m/z value for the peak maximum (midpoint of the line) was calculated after the “deisotoping” step. Small windows of data around the target m/z in the average spectrum were identified, focussing on the M ion rather than M + 1 and M + 2 ions as part of deisotoping the spectra The maximum of the peak was measured and the two closest points to the halfheight on either side were found, resulting in four points. The peak-width calculation (distance of the line) was performed from the points with which a horizontal line at half-height intersected a line connecting the two points on either side of the peak (one above the half-height and one below). For all m/z identity pairs, the maximum intensity was recorded as well as the deviation of the peaks from accurate m/z. This approach has the major advantage that it could be performed independently for each sample. The final step was the combination of all the signals and deviations into their respective files. The technical setup yielded average deviations of less than 4 ppm for the detected lipid species.

**Post-processing.** The peak-picking algorithm initially selected all lipids from a list containing 1305 lipids in positive ionization mode and 3772 lipids in negative ionization mode. These correspond to the expected major ions of all known lipids within the m/z range used and include all the previously identified lipid species from the pooled sample, therefore we refer to it as a semi-targeted approach [14]. QCs were used to remove lipid signals that were not reliably detectable or did not show a linear response. We removed lipid signals present in fewer than 80 % of all QC samples or having a poor correlation with concentration within the dilution range of QC samples (Pearson correlation r < 0.95). We determined the coefficient of variation (CV) for each lipid signal and omitted all lipids with CVs greater than 25 %. For each sample, the sum of the signals of all lipids within each ionization mode that passed the QC steps was calculated. Samples with a total signal for the lipids in a mode lower than than 5 000 000 (relative units) were excluded, as they were considered poor infusion of the sample. Each lipid species was normalized to total area, i.e. expressing it as a proportion of the total signal for each participant. Since most of the lipid signals showed approximate log-normality distribution, we applied natural log-transformation to each normalized lipid signal. We excluded those lipid signals of individual participants whose normalized, log-transformed value was more than 10 standard deviations (SD) from the mean of that lipid across all participants. This was due to the fact that the majority of PROMIS participants were not fasting at time of blood draw, so their lipid levels could have been altered by recently consumed high-fat meals. It is unlikely that lipids would have true values more than 10-SD from the mean, so any excluded measurements were either below the lower limit of detection or the measurement was an artefact, perhaps due to a contaminant.

**Statistical analysis.** Principal components analysis (PCA) was used to estimate the quality of the run and of the processing method and to assess the main differences in lipid profiles across the participants, excluding 17 lipids (3.8 %) with missing signal data in more than 10 % of participants. It was performed within MetaboAnalyst 5.0 [15].

To ensure the best data quality, six internal standards belonging to different lipid classes (0.6 µM 1,2-di-O-octadecyl-snglycero-3-phosphocholine, 1.2 µM 1,2-di-O-phytanyl-sn-glycero-3-phosphoethanolamine, 0.6 µM C8-ceramide, 0.6 µM Nheptadecanoyl-D-erythro-sphingosylphosporylcholine, 6.2 µM undecanoic acid, 0.6 µM trilaurin) were spiked into each sample.

To ensure the best reproducibility across such a big data set, a quality control (QC) sample was created by pooling 100 µL of serum from 200 randomly selected samples, which was mixed and aliquoted for use on each plate. A subset of the QC sample was diluted with phosphate-buffered saline solution to two different concentrations, giving three different QC samples per plate (QC1 was undiluted, QC2 was 1:1 diluted, and QC3 was 1:3 diluted).

To avoid biases from the instrument itself, the running order of the samples was determined according to a randomized block design that was developed using the “blockTools” package [16] in R v3.1.2. Sex, age, ethnicity, centre, and time in years since date of survey were used as factors in the design, and the distance between blocks was minimized for all factors. This ensured the definition of 72 equivalent batches, each batch comprised of eighty extracted blood samples, four blanks and four replicates for each of the 3 QC type (therefore, twelve QC samples per batch).

94 % of samples were missing data from less than 10 % of the 444 lipids, and 96 % of lipids were missing data in less than 10 % of the participants. Only 17 lipids that were missing data in more than 10 % of the participants. All missing values were imputed with the median value for that lipid.

**Fig. 1:**
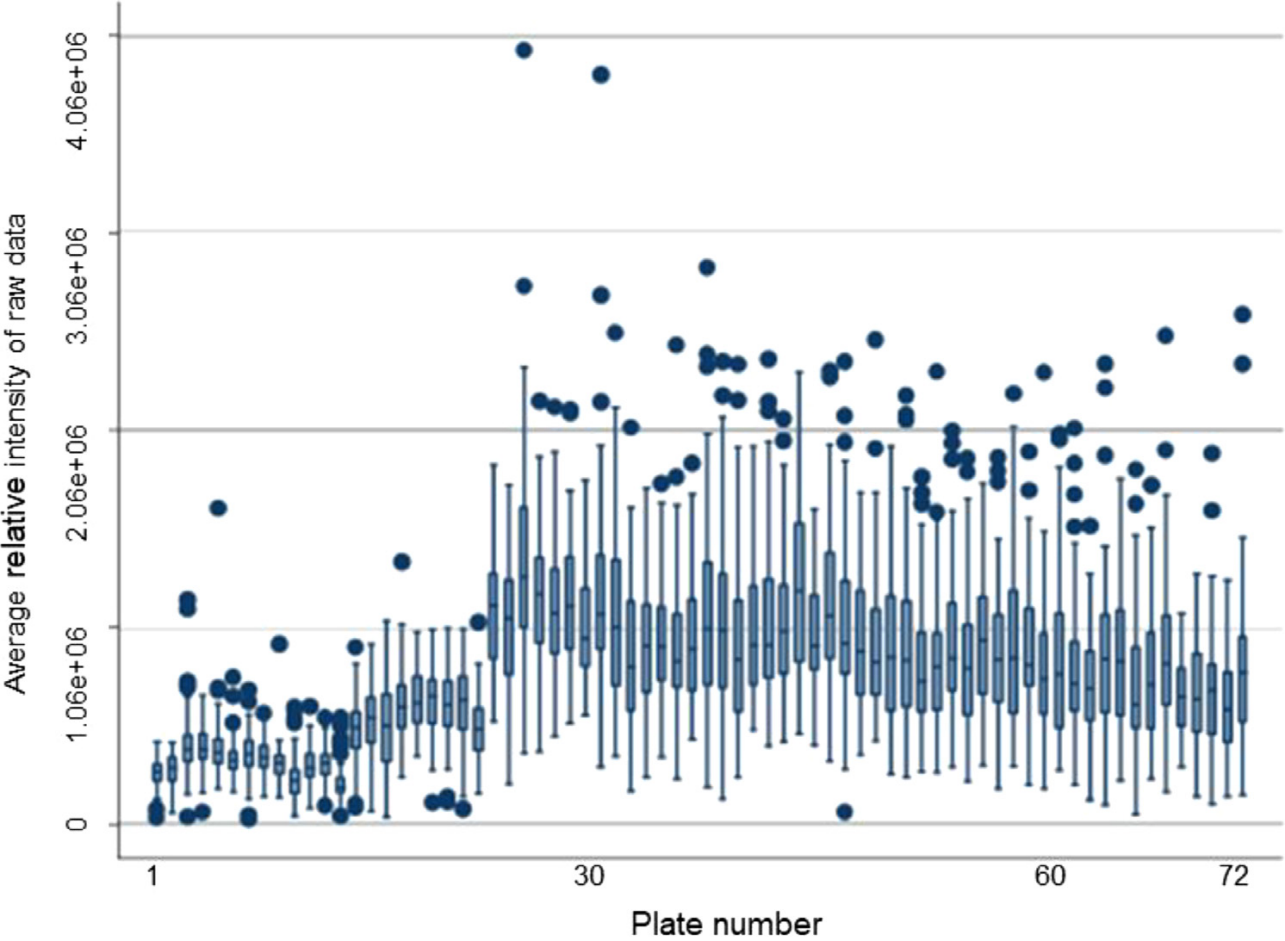
Box plots of raw (pre-normalised) relative intensities of lipids for each participant across each plate.

**Fig. 2:**
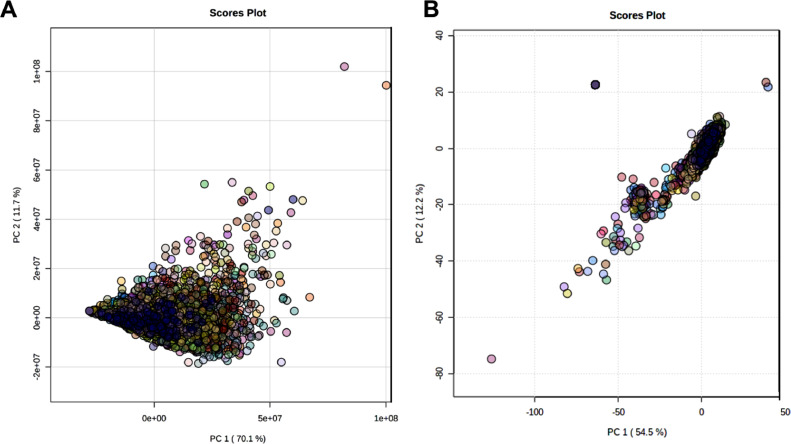
Score plot of first and second principal components of **A.** raw (pre-normalised) and **B.** normalised relative intensities of lipids for each participant for each plate.

**Fig. 3:**
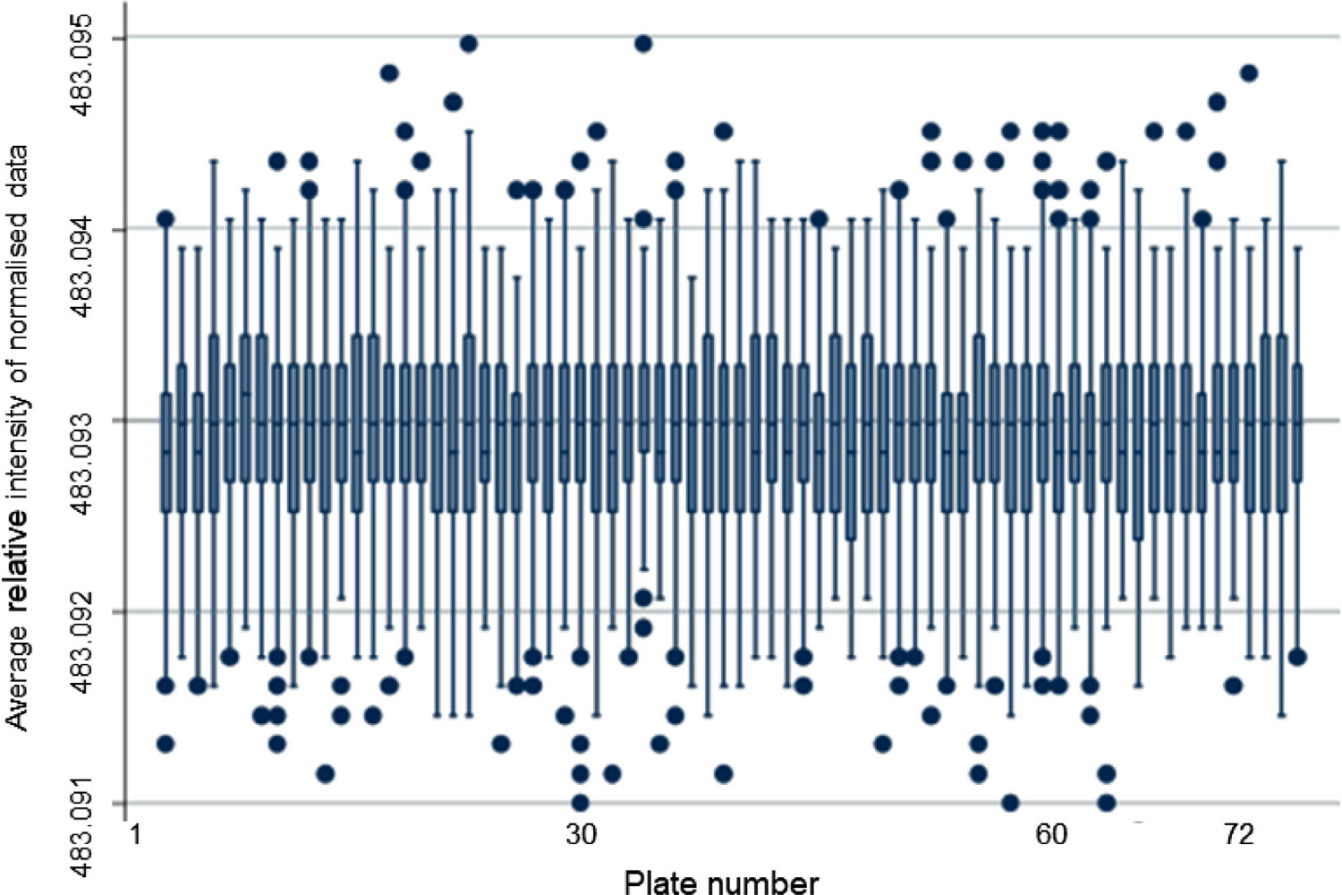
Box plots of normalised relative intensities of lipids for each participant across each plate.

There was a significant observable batch effect (machine drift) in the pre-normalised data across the 72 plates. This was relatively minor for the first few plates but became more pronounced after plate 14 and especially apparent from plate 23 upwards (Fig. 1). It can also be seen in the plot of the first two principal components for each plate (Fig. 2A), together explaining 69 % of the variance in the relative intensities. The cleaning of the instrument heads is likely to be a significant contributor, particularly to the extreme shift in the average relative intensity between plates 22 and 23. However, after normalising the lipids, the batch effect was no longer apparent (Fig. 2B) with the average relative intensity consistent across each plate (Fig. 3).

The CVs for each lipid that were retained in each ionisation mode show that the precision was higher in positive mode (average CV 13.01 %, median CV 11.48 %) than in negative mode (average CV 23.67 %, median CV 22.34 %). However, all this evidence shows that this method gave reproducible data on par with other high-throughput metabolic profiling methods [17].

## Limitations

Our method is unable to separate isobaric species and so would best be described as annotating lipids rather than performing identification of which lipids contribute to a given m/z.

## Ethics Statement

Recruitment of PROMIS participants took place from 2005 to 2011. The study has now completed recruitment, and the final sample size is approximately 16 700 cases and 18 600 controls. PROMIS has been carried out in accordance with the Declaration of Helsinki. The participants were enrolled from the National Institute of Cardiovascular Disorders Karachi, Karachi Institute of Institute of Heart Diseases Karachi, Red Crescent Hospital Hyderabad, Punjab Institute of Cardiology Lahore, Multan Institute of Cardiology Multan, and Faisalabad Institute of Cardiology, Faisalabad. All participants provided written informed consent and the study was approved by the research ethics committee of the Center for Non-Communicable Diseases (CNCD) Pakistan and also by regional Ethical Review committees in the different centres across Pakistan involved in the study. In-addition to the institutional review board (IRB) at CNCD, Karachi, IRBs at National Institute of Cardiovascular Disorders, Karachi, Punjab Institute of Cardiology, Lahore, and Tabba Heart Institute, Karachi approved the study.

## Credit Author Statement

AMW and JLG jointly directed this work. AK generated the lipidomics data. ELH had full access to the data and performed statistical analyses. CC curated the data and submitted it to the repository. CC and JLG drafted the manuscript. All authors contributed important intellectual content to the paper and have given approval to the final version of the manuscript.

## Data Availability

MTBLS4461: An Unbiased Lipid Phenotyping Approach To Study the Genetic Determinants of Lipids and Their Association with Coronary Heart Disease Risk Factors (Original data) (MetaboLights).
